# Protection Conferred by Drinking Water Administration of a Nanoparticle-Based Vaccine against *Salmonella* Enteritidis in Hens

**DOI:** 10.3390/vaccines9030216

**Published:** 2021-03-03

**Authors:** Javier Ochoa-Repáraz, Eduard Sebastià, Marta Sitjà, Ibai Tamayo, Juan Manuel Irache, Carlos Gamazo

**Affiliations:** 1 Department of Microbiology and Parasitology, Universidad de Navarra, 31008 Pamplona, Spain; jochrep@gmail.com (J.O.-R.); ibai.tamayo.rodriguez@navarra.es (I.T.); 2 Department of Biology, Eastern Washington University, Cheney, WA 99004, USA; 3 Hipra Scientific, S.L., 17170 Amer, Spain; eduard.sebastia@hipra.com (E.S.); marta.sitja@hipra.com (M.S.); 4 Department of Chemistry and Pharmaceutical Technology, Universidad de Navarra, 31008 Pamplona, Spain; jmirache@unav.es

**Keywords:** *Salmonella* enteritidis, vaccine, nanoparticles, hens

## Abstract

Salmonellosis remains a major medical and an unmet socioeconomic challenge. Worldwide, more than three million deaths per year are associated with *Salmonella enterica* serovar Enteritidis infections. Although commercially available vaccines for use in poultry exist, their efficacy is limited. We previously described a method for isolating a heat extract (HE) fraction of the cell surface of *S.* Enteritidis that contained major antigenic complexes immunogenic in hens naturally infected with the bacterium. One single dose of S. Enteritidis’ HE induced protection against lethal salmonellosis in mice. Furthermore, HE encapsulation in nanoparticles of the copolymer of methyl vinyl ether and maleic anhydride (PVM/MA), Gantrez AN, improved and prolonged the protection against the disease in mice. We formulated new preparations of Gantrez AN nanoparticles with HE *S.* Enteritidis and assessed their stability in drinking water and their efficacy in hens after experimental infection. The oral treatment of six-week-old hens with two doses of HE nanoparticles significantly reduced the *Salmonella* excretion in hens. Due to the effectiveness of the treatment in reducing bacterial excretion, we conclude that HE nanoencapsulation obtained from S. Enteritidis is a viable novel vaccination approach against salmonellosis in farms.

## 1. Introduction

Infections by *Salmonella enterica* serovar Enteritidis cause one of the most clinically relevant foodborne diseases worldwide [1,2,3]. The foodborne illness caused by *S.* Enteritidis infections accounts for approximately three million deaths per year. In the United States alone, it results in over a million cases per year [4]. As a zoonosis, the bacteria’s reservoir is mostly domesticated fowl and products [1,2,3]. Although the disease often resolves without treatment and antibiotics are effective against the bacterium, vaccination is considered the most effective preventive measure [5]. Although there are available vaccines, their efficacy remains limited. It is generally acknowledged that more effective, stable, and safe vaccines are needed to minimize the socioeconomic and medical burdens of human and poultry salmonellosis [5,6].

In previous works, we described the isolation of a cell surface antigenic extract of *S*. Enteritidis named heat extract (HE). We identified *Salmonella*’s antigenic components, such as lipopolysaccharide, outer-membrane proteins, flagellar epitopes, or fimbriae, as highly reactive against sera obtained from hens during natural infection [7]. Furthermore, we described the protective effects of a single dose of 30 g of HE *S.* Enteritidis against a lethal dose with a pathogenic strain of *S.* Enteritidis in BALB/c mice [8]. The immunological responses triggered by HE were characterized by the induction of T helper 1 (Th1) cell responses [8]. In addition, we performed a proteomics characterization of HE’s antigenic profile and identified cell surface molecules that serve as virulence factors in the pathogenesis of *S.* Enteritidis [9]. Based on our results, we concluded that HE should be considered a promising acellular vaccine candidate.

The commercial vaccines currently available are bacterins, or inactivated vaccines, life bacterial vaccines with attenuated virulence, and subunit vaccines. All three types have limitations worth considering, including poor efficacy, limited cell-mediated or antibody-mediated responses [5,10], and even constitute a hazard in immunocompromised individuals, as might be the case with the life attenuated vaccines [5,10,11,12]. By contrast, subunit vaccines are safer since although they contain antigens, they are unable to replicate. However, multiple doses with these acellular fractions might be needed to confer long-lasting immunity against *Salmonella*’s cell structures, required for protection [13,14,15,16,17,18]. In order to increase the effectiveness of acellular vaccines, the use of adjuvants is extensive. Alternatively, particulate carrier systems such as microparticles and nanoparticles may enhance antigenic mucosal bioavailability even after one single dose. Furthermore, the particles can protect the antigens against degradation through the gastrointestinal tract and can facilitate a sustained release of their cargo, prolonging antigenic exposure. Biodegradable polymers are an economical alternative for mass vaccination in farms due in part to the simple manufacturing protocols. Nanoparticles are formulated with the copolymer of methyl vinyl ether and maleic anhydride (PVM/MA), commercialized as Gantrez AN, as they possess bioadhesive properties [19,20,21]. Gantrez AN nanoparticles have been proposed as mucosal carriers for antigen delivery purposes, including cell surface antigens from *Salmonella* Abortus-ovis [22] and *Salmonella* Enteritidis [9,23,24]. Nanoparticles of Gantrez AN with HE *S*. Enteritidis’ induced protection against lethal salmonellosis in BALB/c mice by stimulating early T helper 1 (Th1) cell responses and subsequent Th2 responses [23].

Here, we characterized the nanoparticles that resulted from HE’s encapsulation obtained from the *S.* Enteritidis strain 449 (NP-HE) in Gantrez AN. We observed that the nanoparticles containing HE were highly stable in drinking water. More importantly, hens’ oral vaccination with our nanoparticle formulations reduced the excretion of a pathogenic strain of *S.* Enteritidis after experimental infection. These studies confirm our previous findings in murine models of lethal infection, indicating the potential protective effects of HE nanoparticles resuspended in drinking water against salmonellosis in hens.

## 2. Materials and Methods

### 2.1. Preparation of Antigenic Extract

Antigenic extracts were obtained from the *Salmonella* Enteritidis strain 449 (Hipra Scientific, S.L.U., Amer, Spain). The strain used in the experimental challenge studies (*S*. Enteritidis LA5) was kindly provided by M.J. Woodward, Veterinary Laboratories Agency, Weybridge, UK. The strains were grown until the stationary phase in a trypticase-soy broth (TSB) (Biomerieux; Marcy L’Etoile, France) on a rotatory shaker at 37 °C for 24 h. After culture, the live cells were harvested by centrifugation and suspended in physiological saline (10 g of packed cells per 100 mL) and heated in flowing steam (100 °C) for 15 min. After centrifugation at 12,000× *g* for 15 min, the supernatant was dialyzed for two days at 4 °C against several changes of deionized water. The dialyzed materials were centrifuged for five hours at 100,000× *g*, and the pellets (HE antigenic extract) were resuspended in deionized H_2_O, lyophilized, and stored at room temperature. The total protein content was determined by a micro bicinchoninic acid (microBCA) protein assay (Pierce, Rockford, CA, USA), with bovine serum albumin as standard.

### 2.2. Preparation of Nanoparticles

Nanoparticles were prepared at a Gantrez AN-to-Salmonella HE ratio of 1.5 (by weight) by a solvent displacement method previously described [9]. For this purpose, 100 mg of Gantrez AN (PVM/MA, Ashland Corp., Barcelona, Spain) were dissolved in 4 mL of acetone. The HE extract was first dispersed in 1 mL of acetone by ultrasonication for 1 min in a MicrosonTM ultrasonicator (Misonix Inc., New York, NY, USA), then incorporated in the polymer solution, and homogenized with shaking for 30 min at 25 °C. The mixtures were poured into a 10-mL stirred ethanol–water phase (1:1, *v*/*v*). The organic solvents were eliminated under reduced pressure (Büchi R-144, Flawil, Switzerland). The resulting nanoparticles were purified by centrifugation at 27,000× *g* for 20 min. The pellets were dispersed in water, and the resulting suspension centrifuged again under the same conditions as described above. The resulting particles were resuspended in an aqueous solution containing 5% lactose before freeze-drying.

### 2.3. Characterization of Nanoparticles

The size of nanoparticles was evaluated by photon correlation spectroscopy using a Zetamaster analyzer system (Malver Instruments Ltd., Worcestershire, UK). The amount of *Salmonella* HE extracts encapsulated in nanoparticles was determined by a microBCA protein assay. Calibration curves were made from the supernatants of blank nanoparticles. Each sample was assayed in triplicate, and results were expressed as HE amounts of protein per mg nanoparticles.

SDS-PAGE of free and nano encapsulated HE extracted from the *S.* Enteritidis strain 449 (NP-HE) was performed as previously described [9] in 12% acrylamide slabs by the method of Laemmli [25], and Coomassie blue was used to stain the gels. A molecular mass marker (Amersham Pharmacia Biotech, Freiburg, Germany) was included to estimate the molecular mass of proteins based on electrophoretic mobility [26].

### 2.4. Analysis of Nanoparticles Stability

Different aqueous solutions were prepared with a different pH and different water hardness. The water total hardness (TH) was expressed as French degree (°fH), since 1 °fH = 4 mg/L Ca^+2^ or 2.43 mg/L Mg^+2^, or CaCO_3_ 10 mg/L. According to the type of water and their characteristics that can be found in many chicken farms, the following aqueous solutions were prepared using CaCL_2_ and MgCL_2_: (i) pH 5.5 with Ca^+2^ and Mg^+2^ (water hardness 30 °fH); (ii) pH 8.5 with Ca^+2^ and Mg^+2^ (water hardness 30 °fH); (iii) Tap water obtained in Pamplona, Spain at pH 6.9 (Water hardness calculated from http://revista.consumer.es; accessed on 27 February 2021); calcium less than 100 mg/L or 20 °fH). Nanoparticles were dispersed in the aqueous media at a concentration of 5 mg/mL, and the turbidity of these suspensions was monitored by spectrophotometry at 405 nm. After four hours, the suspensions were visualized in scanning electron microscopy (SEM) and compared with the initial sample of nanoparticles. The half-life (t ½) of the nanoparticle formulations’ degradation process was estimated from turbidity changes graphs and considered as the time needed to reach 50% of the initial optical density.

### 2.5. Experimental Infections 

Two vaccine doses of NP-HE (experimental vaccine) or Nobilis® Salenvac T (commercial vaccine 1) were administered to hens (at 6 and 9 weeks of age), except for hens that received orally the life vaccine TAD *Salmonella* Vac E^®^ (commercial vaccine 2), that received an initial dose at three days of age. The lyophilized nanoparticles were resuspended in ultrapure, sterile water and administered orally in 3 mL suspensions per hen. The inactivated commercial vaccine 1 was administered intramuscularly (0.5 mL/hen). The attenuated commercial vaccine 2 was administered orally on 3 days, 6 weeks, and 9 weeks of age (1 mL/hen), following the instructions from the manufacturers. Groups of unvaccinated hens received phosphate buffer saline (PBS) as a sham treatment. Three weeks after the last immunization, all hens were experimentally infected with 5 × 10^8^ colony forming units (CFU) of the pathogenic strain *Salmonella* Enteritidis LA5 by oral administration. One week (day 7) and two weeks (day 14) after the experimental infection, the hens were euthanized, and samples of each animal’s cecum, liver, and spleen were aseptically harvested for bacteriological analysis. Cloacal swabs were collected from all hens on days 1, 3, 7, and 14 post-infection.

### 2.6. Animals and Housing Conditions

Specific pathogen-free (SPF) White Leghorn hens were obtained from SPF eggs incubated at Hipra Scientific, S.L.U., Spain. The hens were maintained in an isolated environment until the initiation of the experiments. Each batch of animals was routinely tested serologically to assess *Salmonella* negativity before the beginning of each experiment by bacteriological cultures of cloacal swabs. The birds were kept under regular light and dark cycles and were provided with feed and water ad libitum.

### 2.7. Bacteriological Studies

The tissues were aseptically harvested from hens and placed in a 5 mL sterile saline solution for homogenization. One hundred microliters of the organ suspensions were incubated at 37 °C for 24 h on trypticase–soy agar plates (Biomerieux; Marcy L’Etoile, France) to determine the presence of viable SELA5.

### 2.8. Statistical Analysis

The percentages of isolation of SELA5 in swabs and tissues among the different experimental groups were compared by χ^2^ test. When comparing groups, *p* values below 0.05 were considered statistically significant.

## 3. Results

### 3.1. Characterization of Gantrez AN Nanoparticles with Salmonella HE

**Figure 1:**
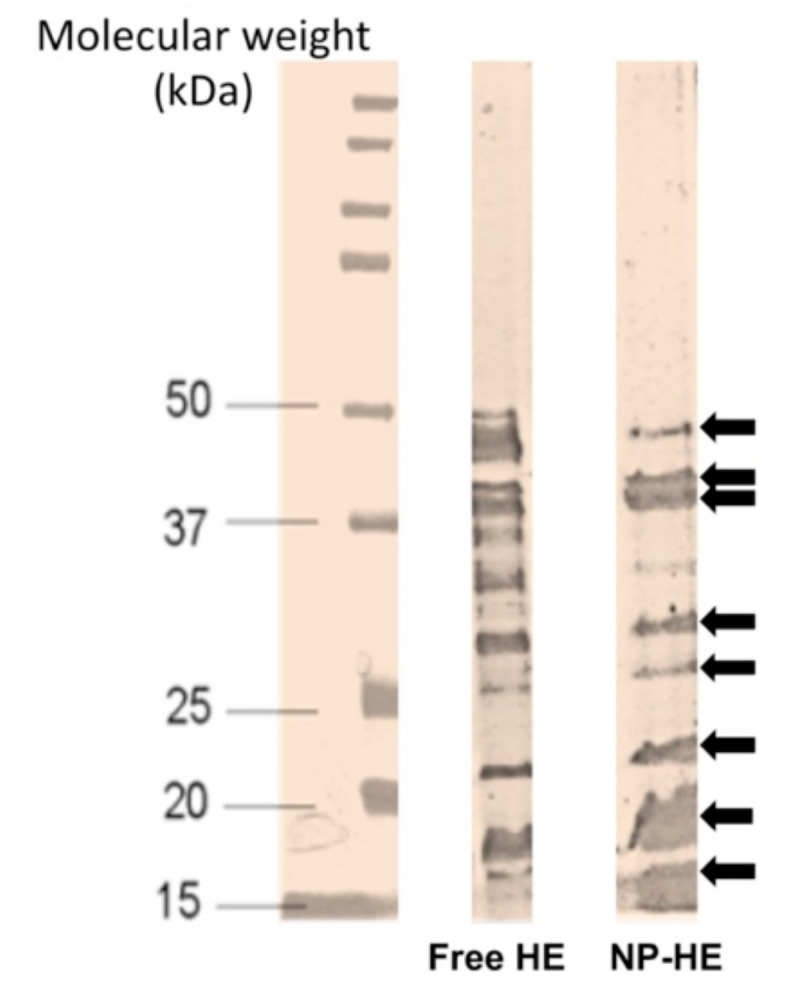
Protein profile of heat extracted (HE) antigenic extract obtained from the *S.* Enteritidis strain 449, free and released in the laboratory from nanoparticles (NP-HE). SDS-PAGE-stained gel with Coomassie blue (image in grayscale; contrasted). Arrows indicate the major well-characterized antigenic proteins with the least reduced integrity after encapsulation (from top to bottom, protein’s molecular weights in kDa: 46.3, 43.5, 41.7, 32.7, 29.0, 22, 17, and 14.5). Lines: molecular weight standard; free HE (40 mg loaded); NP-HE (5 mg).

HE-loaded nanoparticles (NP-HE) presented an average size of about 430 nm with a negative zeta potential of −31 mV. The protein loading was calculated to be 24.1 µg/mg polymer, with an encapsulation efficiency of 72.3%. The encapsulation processes had no effect on the integrity of the main HE proteins; among them, the major well-characterized antigenic proteins had the molecular weights of 46.3, 43.5, 41.7, 32.7, 29, 22, 17, and 14.5 kDa (Figure 1). 

These results were in line with those obtained in our previous publication, indicating that the formulation of HE with the polymer does not affect the antigenic structure of the extracts [9,23]. NP-HE were easily resuspended in tap water without any visible precipitates or agglomerates. Nevertheless, their stability in different aqueous media was evaluated by measuring the turbidity changes of the nanoparticle suspensions as a function of time. This technique, optimized in our labs, correlates the nanoparticles’ concentration and the absorbance change at different wavelengths.

We next compared the stability of NP-HE in the tap water of Pamplona, Spain, and two aqueous solutions with the hardness of 30 °fH and pH of either pH 5.5 or 8.5, respectively, according to the protocol previously described in the methods section. In all cases, NP-HE, independent of the pH and hardness conditions, demonstrated high stability for at least two hours post-incubation in the corresponding solutions. On the contrary, after two hours of incubation in the aqueous media, the nanoparticle dispersions’ turbidity decreased. This decrease was higher when nanoparticles were dispersed in alkaline pH conditions than in acidic ones (Table 1). Thus, the half-life (t ½) of nanoparticles dispersed in water at pH 5.5 was 1 h longer than when dispersed in an aqueous medium at pH 8.5. On the other hand, our results showed that the nanoparticle stability was not affected by a high concentration of divalent cations (calcium or magnesium) (Table 1). This degradation of nanoparticles in an aqueous environment appeared to be mediated by a swelling process, as evidenced by SEM analysis of nanoparticles’ suspensions after four incubation hours in the aqueous media. The polymer Gantrez AN is insoluble in water. However, in an aqueous environment, the anhydride residues would be hydrated and transformed into carboxylic acid groups. In an aqueous environment, particularly under basic conditions, these carboxylic groups would be ionized (negatively charged), inducing a repulsion phenomenon between neighboring carboxylic groups and then facilitating the swelling of nanoparticles [27,28].

**Figure 2:**
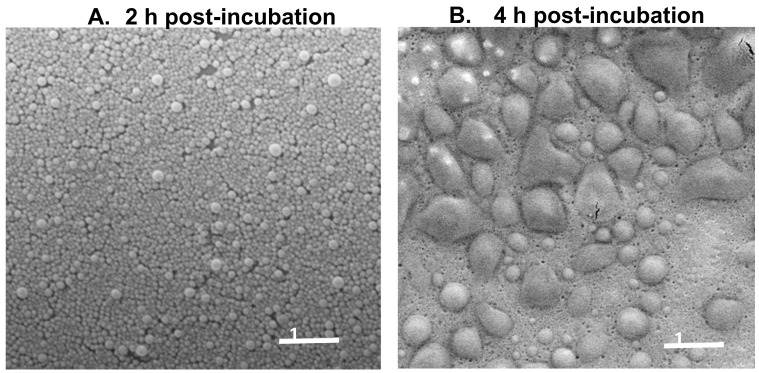
Scanning electron microscopy of nanoparticles containing Salmonella Enteritidis antigenic complex (NP-HE) after the dispersion (**A**) and after 4 h of incubation (**B**) in an aqueous medium at pH 8.5, 30 °fH (Scale bar: 1 µm).

These results were corroborated by scanning electron microscopy to emphasize the nanoparticles’ real degradation (Figure 2). In acidic pH 5.5, all nanoparticle formulations demonstrated a higher stability than in basic pH 8.5. After incubation for 4 h in an aqueous solution, the nanoparticles swelled prior to degradation as shown by electron microscopy (Figure 2).

### 3.2. Gantrez AN Nanoparticles Loaded with Salmonella HE Reduce Bacteria Excretion in Hens

We performed a first protection study comparing the protecting efficacy of NP-HE against experimental infection with the pathogenic strain SELA5 with two commercially available vaccines, the inactivated commercial vaccine 1 and the attenuated commercial vaccine 2. In this first experiment, 28 hens per experimental group were used. As expected, we did not observe any clinical signs compatible with *Salmonella* infection or the control (unvaccinated) group. The necropsies indicated some lesions associated with *Salmonella* infection in some cases, particularly in the liver. No significant differences in the number of lesions were observed among groups (data not shown).

**Figure 3:**
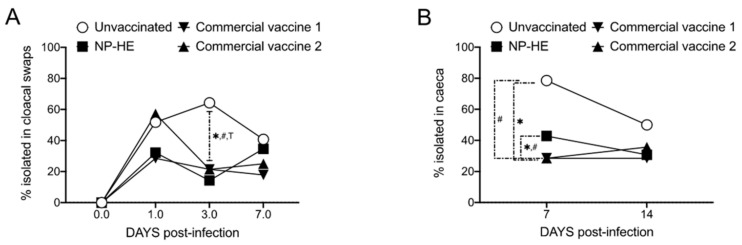

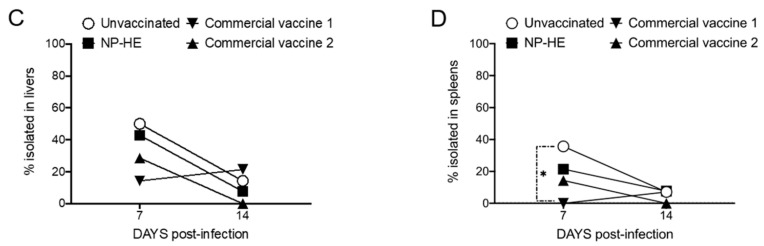
Experimental NP-HE reduces *Salmonella* excretion in hens. Groups of 28 hens were used to compare the efficacy of NP-HE, the inactivated commercial vaccine 1, and the attenuated commercial vaccine 2 against experimental infection with SELA5. Percentages of *Salmonella*-positive samples isolated from cloacal swabs (**A**), ceca (**B**), livers (**C**), and spleens (**D**). The percentages of isolation of *Salmonella* Enteritidis in swabs and tissues among the different experimental groups were compared by χ^2^ test. *, ^#^, and ^T^, *p* < 0.05 for unvaccinated vs. rest of groups.

Infections by *Salmonella* Enteritidis are asymptomatic in immunocompetent hens. The vaccination with commercial vaccine 1, and with the experimental NP-HE resulted in reductions of *Salmonella* excretions (determined in cloacal swabs) through the entire length of the experiment. The vaccination with commercial vaccine 2 also reduced the bacterial excretion but only after day 3 post-infection (Figure 3A). We next compared the percentages of *Salmonella*-positive tissues (ceca, spleen, and livers) isolated from the vaccinated and unvaccinated hens (Figure 3B–D). Overall, we observed a trend of reducing the percentages of *Salmonella*-positive tissues in all tissues isolated from all experimental groups compared with unvaccinated controls. However, the reduction observed was statistically significant only in the ceca of hens administered with the commercial vaccines and in the spleens of hens vaccinated with the commercial vaccine 1, on day 7 post-infection.

**Figure 4:**
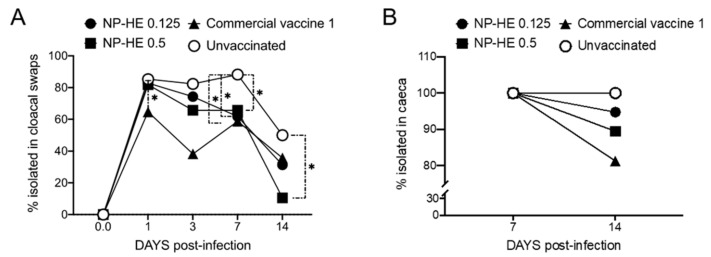

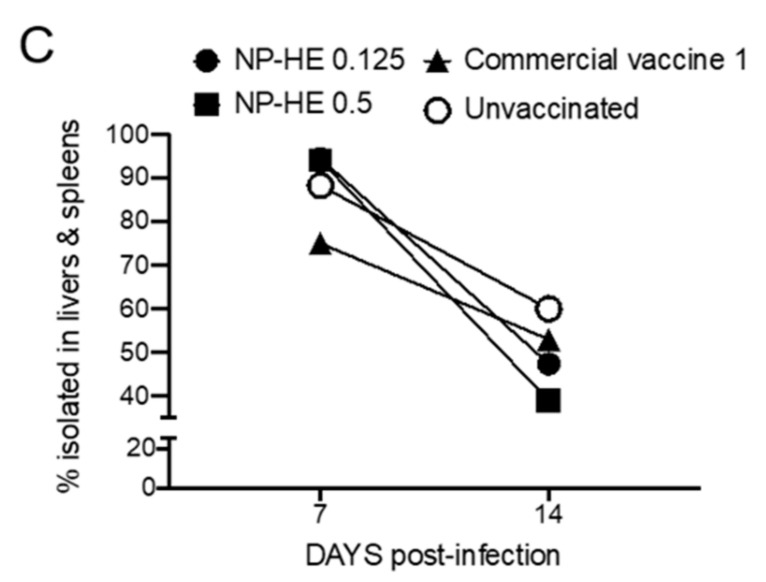
Dose-dependent efficacy of NP-HE experimental vaccines. Groups of 28 hens were used to compare the efficacy of two doses of NP-HE (0.125 mg and 0.5 mg) and the inactivated commercial vaccine 1 against experimental infection with SELA5. Percentages of *Salmonella*-positive samples isolated from cloacal swabs (**A**), ceca (**B**), and a combination of livers and spleens (**C**). The percentages of isolation of *Salmonella* Enteritidis in swabs and tissues among the different experimental groups were compared by χ^2^ test. *, *p* < 0.05.

We next compared the dose-dependent efficacy of NP-HE with the efficacy of the commercial vaccine-1 and unvaccinated hens (Figure 4). Again, 28 hens per experimental group were used. The immunization with the commercial vaccine 1 resulted in a significant reduction in the bacterial excretion on days 1, 3, and 7 post-infection when compared with unvaccinated hens, determined by the bacteriological analysis of the cloacal swabs (Figure 4A). The swabs from hens that received NP-HE (0.125 mg) showed a significant reduction in *Salmonella* excretion on day 7. In comparison, the vaccination with 0.5 mg of NP-HE resulted in significant reductions in excretions on days 7 and 14 compared with the unvaccinated group (Figure 4A). The colonization studies performed in ceca and liver and spleen did not show significant reductions in *Salmonella*-positive tissue percentages. Nevertheless, we observed dose-dependent reductions in the livers and spleens of hens vaccinated with one of the two doses of NP-HE at the end of the experiment (day 14) (Figure 4B,C). In summary, the oral vaccination of 6-week-old SPF hens with the inactivated commercial vaccine 1 was successful in reducing *Salmonella* excretion on days 1, 3, and 7 post-infection. We also observed a significant reduction of *Salmonella* excretion in hens vaccinated with the experimental NP-HE in a dose-dependent manner. The protection of NP-HE appears to be delayed when compared with the commercial vaccine 1.

## 4. Discussion

A controlled delivery of antigens that enhances and prolongs the immune responses is a hallmark of new vaccines’ development. The sustained release of antigens from nanoparticles is a well-described antigen delivery system or approach that can exacerbate the vaccine complex’s immunogenicity [29]. Here, we report that the nanoencapsulation of the heat extracted complex (HE) obtained from *Salmonella Enteritidis* in Gantrez AN nanoparticles reduces bacterial excretion in hens experimentally infected with a pathogenic strain of the bacterium.

Particulate adjuvants are widely evaluated as antigen delivery systems to promote antigenic uptake by antigen-presenting cells that result in balanced cell-mediated and humoral-mediated long-lasting responses against infection [30,31]. We previously showed that HE free from *S.* Enteritidis conferred long-lasting protection in a murine model of salmonellosis [8], suggesting the potential of HEs as vaccine candidates. We were able to identify the antigenic profile of HE by proteomics and defined the presence of cell surface molecules that play significant roles in host–pathogen interactions [9]. We have previously established HE extracts’ immunogenicity using sera from hens [7]. The encapsulation of HE in polymeric delivery systems could enhance the uptake and presentation of the major antigens identified in the extract. Nanoparticles of Gantrez AN, a biocompatible polymer, have been identified as a promising delivery system for drugs [19,20,21], including bacterial extracts isolated from *Salmonella* Abortus-ovis [22] and *S.* Enteritidis [9].

In this work, we performed a characterization of the nanoencapsulation of HE obtained from *S.* Enteritidis 449 (NP-HE) and determined the in vivo efficacy in experiments with hens orally infected with 5 × 10^8^ CFUs of the pathogenic strain of *S.* Enteritidis LA5. The infection does not present clinical signs in hens; however, the animals excrete the bacterium, and internal organ colonization occurs. Our approach consisted of the oral administration of two doses of NP-HE (week 6 and 9 of age) before the oral challenge with the pathogenic strains. We performed stability studies of HE nanoparticle formulations and compared the effects of lactose or mannitol on the stability of NP-HE. Importantly, for commercial purposes, the resuspension of the nanoparticles in tap water (pH 6.9) and acidic and basic solutions demonstrated high stability for at least two hours (Table 1). These results were later confirmed by scanning electron microscopy (Figure 2). These findings are relevant when considering vaccine administration and logistics.

The animals were monitored for two weeks. We performed a bacteriological analysis of cloacal swabs and the cecum, liver, and spleen from each hen to determine the samples’ percentages of *Salmonella*-positivity. We compared our vaccine candidate’s protective effects with two commercially available vaccines, the inactivated commercial vaccine 1 and the attenuated commercial vaccine 2. Our results indicate that NP-HE significantly reduced the excretion of *S.* Enteritidis LA5 in hens in an unknown mechanism that appears to be dose-dependent (Figure 3 and Figure 4). We also observed that the percentages of internal organs of the hens vaccinated with NP-HE positive for *S.* Enteritidis cultures were reduced two weeks after infection. Although no statistical significance was observed in most of the tissues analyzed, the reductions in positive cultures in tissues, and importantly, the significant reduction in bacterial excretion after vaccination with NP-HE suggest that HE nanoparticles could be a novel approach to controlling *Salmonella* spreading in farms. In our protection studies, we used 28 hens per group. More, larger studies are needed in order to determine the appropriate dosing strategy for NP-HE vaccinations.

Both antibody- and cell-mediated responses are needed for protection against *Salmonella* Enteritidis infection [32,33]. In our previous works, we identified the stimulation of early T helper 1 (Th1) cell responses and late increases in serum IgG1 and IgG2a associated with Th2 responses in BALB/c mice protected with Gantrez AN nanoparticles containing HE against lethal challenge with *S.* Enteritidis [9]. The balanced Th1/Th2 responses had previously been documented for particulate adjuvants [21,23,34]. In the BALB/c model, one immunization with Gantrez AN nanoparticles containing HE resulted in 100% protection after two weeks of infection and 80% long-lasting protection against a lethal challenge, outcompeting the commercial vaccines 1 and 2 [9]. Mechanistically, our previous studies in mice indicated that our vaccine candidates promoted a T helper 1 (Th1) response. Serological studies and in vitro approaches are necessary to correlate the mechanism of action observed in the murine models we previously used to test HE and HE nanoparticles and the effects observed in hens.

## 5. Conclusions

The oral administration of two doses of nanoparticles Gantrez AN encapsulating *Salmonella* Enteritidis heat extracts to 6-week-old hens reduces significantly bacterial excretion and appears to reduce bacterial organ colonization. Our studies confirm, in hens, previous results that indicate the potential protective effects of HE nanoparticles against murine infection with *Salmonella* Enteritidis.

## Figures and Tables

**Table 1:** Half-lives (t ½ in min) resulting from the nanoparticles degradation process.

	pH 5.5 ^1^	pH 8.5 ^1^	pH 6.9 (tap) ^2^
NP-HE	202	106	122

^1^ Water hardness 30 °fH. ^2^ Tap water from Pamplona, Spain. Water hardness 20 °fH.

## Data Availability

The data presented in this study are available on request from the corresponding author.
